# Computational Approaches: Drug Discovery and Design in Medicinal Chemistry and Bioinformatics

**DOI:** 10.3390/molecules26247500

**Published:** 2021-12-11

**Authors:** Marco Tutone, Anna Maria Almerico

**Affiliations:** Dipartimento di Scienze e Tecnologie Biologiche Chimiche e Farmaceutiche (STEBICEF), Università degli Studi di Palermo, Via Archirafi 32, 90123 Palermo, Italy; annamaria.almerico@unipa.it

To date, computational approaches have been recognized as a key component in drug design and discovery workflows. Developed to help researchers save time and reduce costs, several computational tools have been developed and implemented in the last twenty years. At present, they are routinely used to identify a therapeutic target, understand ligand–protein and protein–protein interactions, and identify orthosteric and allosteric binding sites, but their primary use remains the identification of hits through ligand-based and structure-based virtual screening and the optimization of lead compounds, followed by the estimation of the binding free energy. The repurposing of an old drug for the treatment of new diseases, helped by in silico tools, has also gained a prominent role in virtual screening campaigns.

Moreover, the availability and the decreasing cost of hardware and software, together with the development of several web servers on which to upload and download computational data, have contributed to the success of computer-assisted drug design. These improved, accurate, and reliable methods should help to add new and more potent molecules to the paraphernalia of approved drugs. Nevertheless, the ease of access of computational tools in drug design (software, databases, libraries, and web servers) should not encourage users with little or almost no knowledge of the underlying physical basis of the methods used, who could compromise the interpretation of the results. The figure of the computational (medicinal) chemist should be recognized and included in all research groups. These considerations led us to promote a volume collecting some original contributions regarding all aspects of the computational approaches, such as docking, induced-fit docking, molecular dynamics simulations, free energy calculations, and reverse modeling. We also include ligand-based approaches, such as molecular similarity fingerprints, shape methods, pharmacophore modeling, and QSAR. Drug design and the development process strive to predict the metabolic fate of a drug candidate to establish a relationship between the pharmacodynamics and pharmacokinetics and highlight the potential toxicity of the drug candidate. Even though the use of computational approaches is often combined, we tried to identify which of these play a central role in each manuscript.

In this Special Issue, the use of molecular dynamics simulations, both unbiased and biased, cover a major part of the contributions. Non-covalent inhibition of the immunoproteasome was investigated in-depth through MD-binding and binding pose metadynamics [1]. MD simulations provided insight into the structural features of hTSPO (Translocator Protein) and the previously unknown interplay between PK11195, a molecule routinely used in positron emission tomography (PET) for the imaging of neuroinflammatory sites, and cholesterol [2]. The interaction of certain endogen substrates, drug substrates, and inhibitors with wild-type MRP4 (WT-MRP4) and its variants G187W and Y556C were studied to determine differences in the intermolecular interactions and affinity related to SNPs using several approaches, but particularly all-atom, coarse-grained, and umbrella sampling molecular dynamics simulations (AA-MDS and CG-MDS, respectively) [3]. Natural sodium–glucose co-transporter 2 (SGLT2) inhibitors were selected to explore their potential against an emerging uropathogenic bacterial therapeutic target, i.e., FimH, which plays a critical role in the colonization of uropathogenic bacteria on the urinary tract surface, and molecular dynamics simulations were carried out to study the potential interactions [4]. Doxorubicin encapsulation in carbon nanotubes with haeckelite or Stone–Wales defects as drug carriers were investigated using a molecular dynamics approach [5]. The combined use of different approaches has been reported in a series of papers associated with the virtual screening of libraries. Almeelebia and co. screened 224,205 natural compounds from the ZINC database against the catalytic site of the Mtb proteasome [6]. Pharmacophore-based virtual screening and molecular docking were carried out to identify potential Src inhibitors starting from a total of 891 molecules. Finally, MD simulations identified two molecules as potential lead compounds against Src kinase [7]. An in silico study identified a methotrexate analog as a potential inhibitor of drug-resistant human dihydrofolate reductase for cancer therapeutics [8]. A structure-based method for high-throughput virtual screening aimed to identify new dual-target hit molecules for acetylcholinesterase, and the α7 nicotinic acetylcholine receptor was reported and confirmed in vitro [9]. A new complementary computational analysis called “dock binning” evaluates antibody–antigen docking models to identify why and where they might compete in terms of possible binding sites on the antigen [10]. Interesting drug repurposing strategies have been reported. Hudson and Samudrala presented a computational analysis of a novel drug opportunities (CANDO) platform for shotgun multitarget repurposing. It implements several pipelines for the large-scale modeling and simulation of interactions between comprehensive libraries of drugs/compounds and protein structures [11]. Qi and co. data-mined the crowd extracted expression of differential signatures (CREEDS) database to evaluate the similarities between gene expression signature (GES) profiles from drugs and their indicated diseases for GES-guided drug-repositioning approaches [12]. In late 2019, the SARS-CoV-2 pandemic focused the attention of many researchers intending to find not only vaccines but also new antiviral drugs. These reasons boosted the use of computational approaches to explore large libraries of natural compounds, already approved drugs, and in-house and commercial compounds [13,14]. In this issue, Baig and co. studied the efficacy of the Mpro inhibitor PF-00835231 against Mpro and its reported mutants in clinical trials. Several in silico approaches were used to investigate and compare the efficacy of PF-00835231 and five drugs previously documented to inhibit Mpro [15]. Li and co. computationally investigated the MPD3 phytochemical database along with the pool of reported natural antiviral compounds to be used against SARS-CoV-2 [16]. Pedretti and co., exploiting the availability of resolved structures, designed a structure-based computational approach. The innovative idea of their study was to exploit known inhibitors of SARS-CoV 3CL-Pro as a training set to perform and validate multiple virtual screening campaigns [17]. In the context of antiviral drugs, Regad and co. investigated the emergence of HIV-2 resistance. They proposed a structural analysis of 31 drug-resistant mutants of HIV-2 protease (PR2), an important target against HIV-2 infection [18]. A wide series of contributions regarding the use of QSAR, machine learning, and deep learning has reported interesting outcomes. A multiple-molecule drug design based on systems biology approaches and a deep neural network to mitigate human skin aging was developed by Yeh and co. With the proposed systems medicine design procedure, they not only shed light on the skin-aging molecular progression mechanisms, but they also suggested two multiple-molecule drugs to mitigate human skin aging [19]. The construction of quantitative structure–activity relationship (QSAR) models was used to predict the biological activities of the compounds obtained with virtual screening and identify new selective chemical entities for the COX-2 enzyme [20]. The three-dimensional QSAR model, employing a common-features pharmacophore as an alignment rule, was built on 20 highly active/selective HDAC1 inhibitors. The predictive power of the 3D QSAR model represents a useful filtering tool for screening large chemical databases, finding novel derivatives with improved HDAC1 inhibitory activity [21]. Different machine learning (ML) and deep learning (DL) algorithms using various integer and binary type fingerprints were evaluated to develop quantitative structure–activity relationship (QSAR) models, which are important for hERG potassium channel blocker prediction [22].

Throughout this Special Issue, all the recent aspects of the computational approaches applied to several research fields are reported. We express our deep gratitude to all the contributors to this Special Issue for their commitment, hard work, and outstanding papers. We also thank all the reviewers involved in the manuscript revisions for their unpaid contributions to improve any aspects of the submitted works. Last but not least, we deeply thank Mrs. Jessie Zhang for her assistance during the period in which we served as guest editors.
